# Estimating the direct healthcare costs of oral diseases in the United Kingdom: a cost-of-illness projection to 2050

**DOI:** 10.3389/fpubh.2026.1863227

**Published:** 2026-06-12

**Authors:** Edward Coote, Nitesh Patel, Rajesh Vijayanarayanan

**Affiliations:** 21D Clinical Limited, Warrington, United Kingdom

**Keywords:** cost-of-illness (COI), healthcare spending, NHS (National Health Service), oral health, periodontal disease

## Abstract

**Aims:**

This study aims to estimate the annual NHS treatment cost associated with projected dental caries and periodontal disease burden in the UK adult population from 2020 to 2050.

**Methods:**

A prevalence-based cost-of-illness projection was built from population-level estimates of dental caries, periodontal pocketing and loss of attachment from a previously published multi-state population projection model. Bottom-up costing from an NHS perspective used the Units of Dental Activity contract framework with attendance rates derived from the adult oral health survey.

**Results:**

Total annual NHS costs are projected to rise from £4,418 m in 2020 (95% CI £4,388–£4,449 m) to £5,301 m in 2050 (£5,204–£5,398 m), a 20% increase. The 60+ age group will bear 69% of caries-related costs by 2050, up from 45% in 2020. Untreated caries costs in those aged 60 + will grow by 168%, and severe periodontal pocketing costs by 57%. Per capita cost rises from £80.76 to £90.84.

**Conclusion:**

NHS dental expenditure will be increasingly concentrated among older adults with severe disease. These findings provide an economic rationale for focusing NHS dental commissioning towards older adult oral health and earlier-life prevention.

## 1 Introduction

Oral diseases, including periodontal disease, tooth loss and dental caries, are prevalent chronic diseases. The global oral disease burden is projected to grow substantially owing to an increasingly ageing population, disease chronicity and healthcare disparities between countries (1). In the UK, untreated caries and severe periodontal pocketing are estimated to increase by 75.2% and 56.7%, respectively, between 2020 and 2050 (2). From the most recent Adult Oral Health Survey (AOHS) in 2023, 21% of dentate adults have at least one tooth with extensive obvious decay, and 49% reported that they had experienced an occasional or frequent oral health impact (3). The AOHS reported that 28% of individuals had periodontal pocketing greater than 3.5 mm, using the Basic Periodontal Examination severity criteria used in UK Dentistry (4).

The economic burden of poor oral health on health systems is expected to be substantial. The most recent estimate of total dental health expenditure in the UK was estimated at €3.52 billion in 2018, representing 1.47% of total health expenditure (5). Such economic estimates are essential in aiding policy planning, however, detailed national-level projections estimating future health system costs for the NHS are sparse in the oral health literature. Based on Global Burden of Disease estimates the global direct and indirect costs of dental diseases are $356.80 billion and $187.61 billion, respectively, but without age- or severity-stratified UK-level estimates there is an absence of reliable economic burden estimates (6). This limits the policy relevance of this projection, as action plans will require a specific level of granularity to support specific policy targetting.

Cost-of-illness (COI) methods using national-level parameters have, to our knowledge, yet to be applied to oral diseases in the UK. They have been commonly applied to other chronic diseases, such as cardiovascular disease, and have become a useful tool to encourage policy debate and measure resource use impact (7, 8). COI projections in these chronic disease spaces have been able to quantify the economic burden of disease, which is required in the context of oral diseases in the UK given an absence of one. It is distinct from cost-effectiveness analysis, which compares multiple interventions, and budget impact analysis, which measures the financial impact of a specific decision. This study estimates the annual NHS treatment cost associated with projected dental caries and periodontal disease burden in the UK adult population from 2020 to 2050.

## 2 Materials and methods

A prevalence-based COI projection with population-level estimates of dental caries, periodontal pocketing and loss of attachment (LOA) from a previously published multi-state population projection was used to model costs in constant prices associated with dental caries and periodontal disease in the UK adult population (2). Bottom-up costing was applied using an NHS perspective, using the Units of Dental Activity (UDA) contract framework with attendance rates derived from the AOHS 2023 (3).

Productivity losses were excluded due to insufficient state-specific UK data (9). Elamin and Ansah only provide prevalence estimations at three time points (2020, 2035, 2050) so we had to interpolate linearly between 2020–2035 and 2035–2050 to produce disease prevalence estimates for each year. This assumes smooth disease prevalence flows between the provided years.

An effective annual cost per person in each state was calculated using the product of: (1) the cost per course of treatment derived from the UDA contract value and the number of UDAs per treatment band, allocated to each disease state based on the expected pathway, (2) the annual attendance rate from the AOHS using age group splits for caries states and the overall rate for periodontal states, (3) the expected number of courses of treatment per attendee per year reflecting the severity of disease. Urgent care module costs for each disease state were calculated using the emergency presentation rate given in the AOHS for caries, and assumed rates for periodontal states using clinical judgement, multiplied by the urgent care cost per event (which was calculated as 1.2 UDAs) (Supplementary Table S1). The average UDA contract value (£31) was provided by a Healthcare Financial Management Association (HFMA) 2025 report (10). This was provided as the mean contract value across NHS dental practices in England, but was varied in sensitivity analysis using a range of £28–£45, which reflects the UDA minimum and maximum reported contract price. The NHS dental band structure and UDA weightings were sourced from the NHS Business Services Authority (11, 12). A weighted average across sub-bands (Band 2a, 2b, 2c) was used where necessary. Costs are reported in constant 2024/2025 GBP (£). Untreated caries refers to individuals with visual and/or cavitated caries, unrestorable teeth or recurrent caries on restorations (2). No caries population was used for check up costs of orally healthy individuals, no periodontal pocketing was not included in total costs in order to not double count (2). The projected costs will not include direct NHS costs for related conditions such as oral cancers or paediatric care. Full state-by-state costs can be found in Table 1, with full justification for attendance and effective annual state-by-state cost per person in Supplementary Table S1.

**Table 1:** Summary of total annual direct costs of oral diseases state-by-state.

Disease state	Cost per COT (£)	Effective annual cost (£)	Source
Caries states			
No caries	31.00	15.81	(3, 10–12)
Untreated caries 16–24	120.90	62.99	(3, 10–12); COTs/yr. assumed
Untreated caries 25–59	106.95	55.61	(3, 10–12); COTs/yr. assumed
Untreated caries 60+	162.75	110.67	(3, 10–12); COTs/yr. assumed
Treated caries 16–24	31.00	16.15	(3, 10–12)
Treated caries 25–59	31.00	16.12	(3, 10–12)
Treated caries 60+	31.00	21.08	(3, 10–12)
Periodontal states			
No periodontal pocketing	31.00	17.98	(3, 10–12)
Mild pocketing (4–<6 mm)	93.00	53.94	(3, 10–12); COTs/yr. assumed
Moderate pocketing (6–<9 mm)	93.00	53.94	(3, 10–12); COTs/yr. assumed
Severe pocketing (≥9 mm)	232.50	269.70	(3, 10–12); COTs/yr. assumed
LOA states			
Mild LOA (4–<6 mm)	93.00	63.24	(3, 10–12); COTs/yr. assumed
Moderate LOA (6–<9 mm)	93.00	63.24	(3, 10–12); COTs/yr. assumed
Severe LOA (≥9 mm)	232.50	316.20	(3, 10–12); COTs/yr. assumed

Effective annual cost per person was calculated by multiplying cost per course of treatment by the expected attendance rate and the courses of treatment per attendee/yr expected in that state. UDA contract value from HFMA (10); NHS dental band UDA weightings from NHS Business Services Authority (11, 12); attendance rates from Adult Oral Health Survey 2023 (3); courses of treatment per attendee per year derived from clinical pathway assumptions (see Supplementary Table S1).

Top-down validation was used to check the magnitude of our aggregate estimates against published NHS dental expenditure. NHS England reported total dental spending of £3.72 billion in 2024/25, and we estimated a total annual spending of £4.58 billion for the whole UK (13). Scaling our whole UK estimate using England’s population share (84%) gives £3.85 billion, signalling an overestimation of 3.5% which we attribute to our model’s assumption of full UDA delivery against contract targets (see Table 2).

**Table 2:** Parameter ranges used in one-way sensitivity analysis.

Parameter	Base case	Low value	High value
Cost drivers			
UDA contract value (£)	31.00	28.00	45.00
Untreated caries 16–24: cost/COT (£)	120.90	93.00	162.75
Untreated caries 25–59: cost/COT (£)	106.95	93.00	162.75
Untreated caries 60+: cost/COT (£)	162.75	93.00	232.50
Severe pocketing: cost/COT (£)	232.50	93.00	372.00
Severe pocketing: COTs per year	2	1	3
Severe LOA: cost/COT (£)	232.50	93.00	372.00
Attendance rates			
Attendance rate: 16–24	52.1%	38.5%	63.9%
Attendance rate: 25–59	52.0%	40.0%	65.0%
Attendance rate: 60+	68.0%	50.0%	80.0%
Attendance rate: periodontal (overall)	58.0%	40.0%	73.0%
Attendance rate: LOA (60+)	68.0%	50.0%	80.0%
No caries/No periodontal attendance rate	51.0%	35.0%	65.0%
Urgent care rates			
Urgent rate: untreated caries 25–59	20.0%	10.0%	30.0%
Urgent rate: untreated caries 60+	22.0%	11.0%	33.0%
Urgent rate: severe pocketing	15.0%	5.0%	25.0%
All urgent rates (× multiplier)	1.0	0.5	2.0

Each parameter was varied independently to its low and high value while holding all other parameters at base case. COT, course of treatment; UDA, unit of dental activity; LOA, loss of attachment.

To address the uncertainty in our model, confidence intervals (CIs) provided by Elamin and Ansah from the source population prevalence model were used to produce upper and lower limits of our estimations (2). This tests the uncertainty arising from epidemiological factors in our calculations. One-way sensitivity analysis captured cost-side uncertainties in the UDA value, attendance rates and disease state costs. Each parameter was varied using its upper and lower limits while holding all others at base case value.

## 3 Results

**Figure 1:**
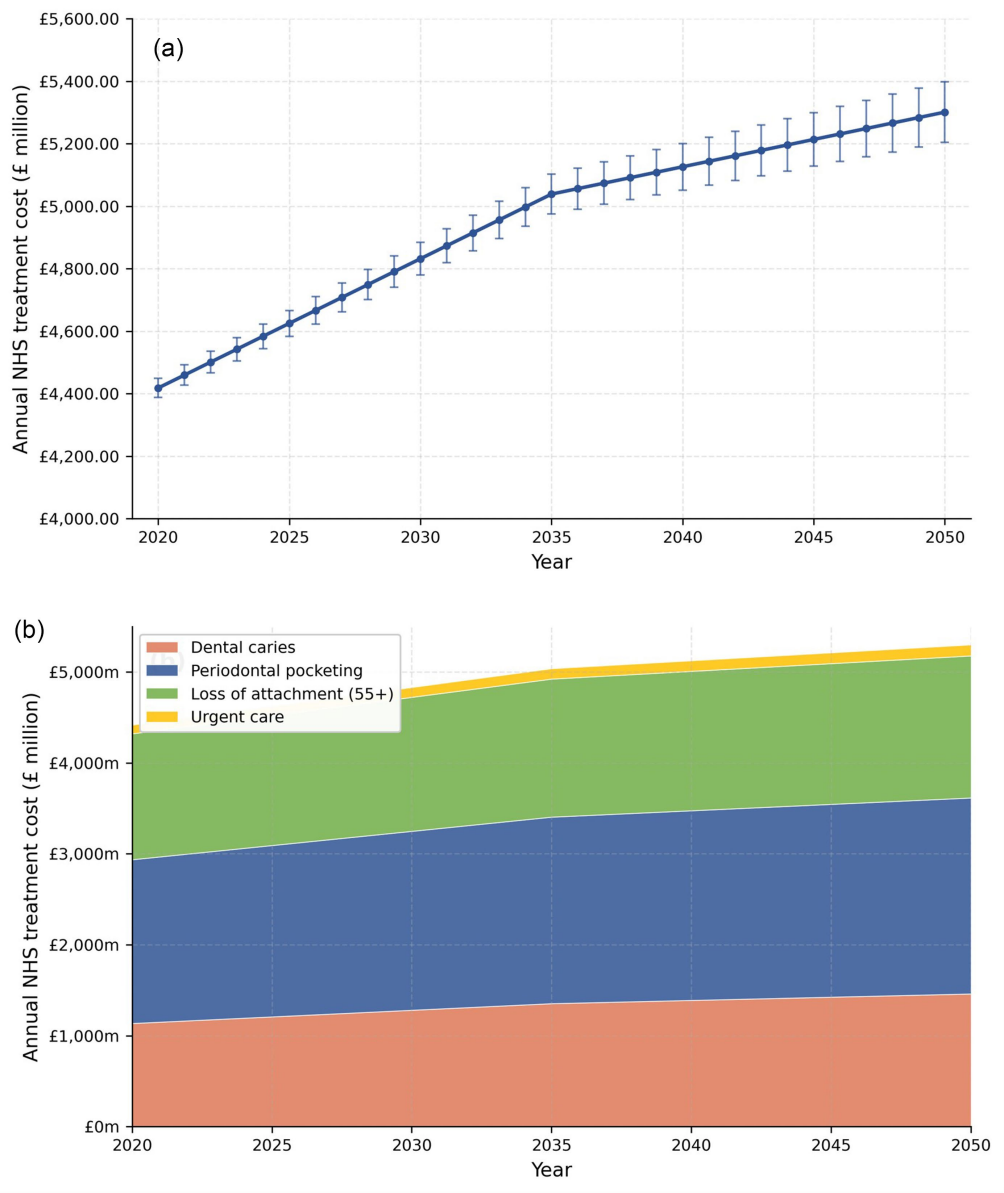
**(a)** Projected annual cost to the NHS of oral diseases 2020–2050 (£m); **(b)** Projected NHS treatment costs by disease component 2020–2050 (£m).

Table 3 shows the results of the projected COI of dental caries and periodontal disease in the UK between 2020 and 2050. The total cost to the NHS of treating dental caries, periodontal disease and LOA is projected to increase from £4,418 m in 2020 (95% CI £4,387 m–£4,448 m) to £5,300 m in 2050 (£5,203 m–£5,397 m) (Figure 1a), which represents a 20% increase. Urgent care costs are expected to grow by 26% (25%–28%) between 2020 and 2050, which makes up 2% of the total costs in 2050 (£121.65 m; 95% CI £118.89 m–£124.40 m). Urgent events from untreated caries for those aged 60 and over are projected the largest growth in this sub-category, resulting in the largest costs in this category in 2050 (£30.73 m; 95% CI £30.17 m–£31.29 m) and a growth rate of 168% (167%–170%) over the forecast period.

**Table 3:** Projected annual cost of oral health diseases in the UK from 2020 to 2050 (95% CI).

Category	2020	2025	2030	2035	2040	2045	2050
Total annual cost of illness (£m)	£4,418 m (4,388–4,449)	£4,625 m (4,583–4,666)	£4,832 m (4,779–4,884)	£5,039 m (4,975–5,102)	£5,126 m (5,051–5,201)	£5,213 m (5,128–5,299)	£5,301 m (5,204–5,398)
Caries costs	£1,135 m (1,131–1,139)	£1,208 m (1,202–1,214)	£1,281 m (1,273–1,289)	£1,354 m (1,343–1,364)	£1,389 m (1,376–1,403)	£1,425 m (1,408–1,442)	£1,461 m (1,440–1,481)
Periodontal costs	£1,801 m (1,786–1,817)	£1,885 m (1,863–1,906)	£1,968 m (1,941–1,996)	£2,052 m (2,018–2,085)	£2,086 m (2,046–2,126)	£2,121 m (2,075–2,167)	£2,156 m (2,103–2,208)
Urgent care costs	£96.3 m (95.5–97.0)	£102.3 m (101.2–103.4)	£108.3 m (106.9–109.7)	£114.4 m (112.6–116.1)	£116.8 m (114.7–118.9)	£119.2 m (116.8–121.6)	£121.7 m (118.9–124.4)
Cost per capita (UK adult)	£80.76 (80.20–81.32)	£82.75 (82.01–83.49)	£84.66 (83.74–85.58)	£86.49 (85.40–87.58)	£87.94 (86.66–89.22)	£89.39 (87.92–90.86)	£90.84 (89.17–92.49)
Age-band caries							
Treated & untreated – 16–24 yrs	£14.3 m (14.0–14.6)	£13.9 m (13.5–14.3)	£13.4 m (12.9–13.9)	£13.0 m (12.4–13.5)	£12.7 m (12.1–13.3)	£12.5 m (11.9–13.1)	£12.3 m (11.6–12.9)
Treated & untreated – 25–59 yrs	£272.6 m (270.5–274.6)	£270.2 m (267.5–273.0)	£267.9 m (264.4–271.3)	£265.5 m (261.3–269.7)	£254.2 m (249.1–259.3)	£242.9 m (236.9–248.9)	£231.6 m (224.7–238.6)
Treated & untreated – 60+ yrs	£232.4 m (230.3–234.5)	£292.5 m (289.3–295.8)	£352.7 m (348.3–357.1)	£412.8 m (407.3–418.4)	£455.0 m (448.4–461.5)	£497.1 m (489.6–504.5)	£539.3 m (530.8–547.6)
% Of total borne by 60+	44.8% (44.7–44.8)	50.7% (50.7–50.7)	55.6% (55.7–55.6)	59.7% (59.8–59.6)	63.0% (63.2–62.9)	66.1% (66.3–65.8)	68.9% (69.2–68.5)
Pocketing by severity							
Mild (4–<6 mm)	£1,115 m (1,109–1,122)	£1,143 m (1,134–1,153)	£1,171 m (1,159–1,184)	£1,199 m (1,184–1,215)	£1,204 m (1,186–1,222)	£1,209 m (1,187–1,230)	£1,213 m (1,189–1,238)
Moderate (6–<9 mm)	£171 m (170–171)	£165 m (164–166)	£160 m (159–161)	£155 m (153–156)	£148 m (146–150)	£141 m (139–143)	£134 m (132–137)
Severe (≥9 mm)	£516 m (508–523)	£576 m (565–587)	£637 m (623–651)	£698 m (680–715)	£734 m (715–754)	£771 m (749–794)	£808 m (783–833)
% Borne by severe	28.6% (28.4–28.8)	30.6% (30.3–30.8)	32.4% (32.1–32.6)	34.0% (33.7–34.3)	35.2% (34.9–35.5)	36.4% (36.1–36.6)	37.5% (37.2–37.7)
Loss of attachment (LOA)							
Mild (4–<6 mm)	£854 m (850–858)	£886 m (883–890)	£919 m (915–923)	£951 m (947–955)	£964 m (960–968)	£977 m (973–982)	£991 m (986–995)
Moderate (6–<9 mm)	£275 m (272–279)	£276 m (271–281)	£277 m (271–283)	£278 m (271–285)	£276 m (268–283)	£273 m (265–281)	£271 m (262–279)
Severe (≥9 mm)	£256 m (252–259)	£267 m (263–272)	£278 m (273–284)	£290 m (283–297)	£294 m (286–301)	£298 m (290–305)	£302 m (293–310)
% Borne by severe	18.5% (18.4–18.6)	18.7% (18.5–18.8)	18.9% (18.7–19.1)	19.1% (18.9–19.3)	19.1% (18.9–19.4)	19.2% (19.0–19.5)	19.3% (19.0–19.6)
Urgent care							
Untreated caries 16–24	£0.33 m (0.33–0.34)	£0.34 m (0.32–0.34)	£0.34 m (0.32–0.35)	£0.34 m (0.32–0.35)	£0.33 m (0.32–0.35)	£0.32 m (0.31–0.34)	£0.32 m (0.30–0.34)
Untreated caries 25–59	£20.0 m (19.7–20.2)	£21.7 m (21.3–22.0)	£23.3 m (22.9–23.7)	£25.0 m (24.5–25.5)	£25.5 m (24.9–26.1)	£26.0 m (25.3–26.7)	£26.5 m (25.7–27.3)
Untreated caries 60+	£11.4 m (11.3–11.6)	£15.2 m (14.9–15.4)	£18.9 m (18.6–19.2)	£22.6 m (22.2–22.9)	£25.3 m (24.9–25.7)	£28.0 m (27.5–28.5)	£30.7 m (30.2–31.3)
Treated caries (all)	£21.3 m (21.3–21.4)	£20.4 m (20.4–20.5)	£19.6 m (19.5–19.6)	£18.7 m (18.6–18.8)	£17.4 m (17.3–17.5)	£16.2 m (16.0–16.3)	£14.9 m (14.7–15.1)
Mild pocketing	£23.1 m (22.9–23.2)	£23.7 m (23.5–23.9)	£24.2 m (24.0–24.5)	£24.8 m (24.5–25.1)	£24.9 m (24.5–25.3)	£25.0 m (24.6–25.5)	£25.1 m (24.6–25.6)
Moderate pocketing	£9.4 m (9.4–9.4)	£9.1 m (9.1–9.2)	£8.8 m (8.8–8.9)	£8.5 m (8.4–8.6)	£8.2 m (8.1–8.3)	£7.8 m (7.7–7.9)	£7.4 m (7.3–7.6)
Severe pocketing	£10.7 m (10.5–10.8)	£11.9 m (11.7–12.1)	£13.2 m (12.9–13.5)	£14.4 m (14.1–14.8)	£15.2 m (14.8–15.6)	£16.0 m (15.5–16.4)	£16.7 m (16.2–17.2)

Direct NHS costs related to dental caries have been projected to grow the largest out of the sub-categories, with a 29% growth rate from 2020 to 2050, resulting in £1,461 m (£1,440 m–£1,481 m) in 2050. Untreated caries in those aged 60 years and over is projected to reach £415.57 m (£407.93 m–£423.09 m), which has the largest estimated growth rate over the period at 168% (167–170%). Check-up costs for the caries-free population represent the largest single component of caries expenditure, estimated at £677.51 m in 2050, owing to the continuously rising population in the model. The costs associated with individuals with untreated caries aged 16–24, and those aged 16–24 and 25–59 with treated caries are all projected to decrease between 2020 and 2050.

Costs related to periodontal pocketing are expected to remain the largest throughout the forecast period (Figure 1b). Total costs related to periodontal pocketing are estimated to reach £2,156 m (£2,103 m–£2,208 m) in 2050, representing a 20% (18%–22%) increase. Although mild pocketing is projected to be the severity stage that comprises the most costs, with £1,213.38 m (£1,188.62 m–£1,238.14 m) by 2050, and a 9% (7%–10%) increase from 2020 to 2050 was estimated. The costs associated with severe pocketing will increase by 57% (54%–59%) over the forecast period, reaching £808.02 m (£782.67 m–£833.37 m) in 2050. Alongside this, we estimate that the direct costs of moderate pocketing will decrease by 21% ( − 23% to − 21%), remaining the smallest cost of the pocketing severities at £134.26 m (£131.61 m–£136.85 m) in 2050.

Costs related to treating LOA are projected to reach £1,562.91 m (£1,541.79 m–£1,583.91 m) in 2050, which is a 13% increase (12%–14%) from 2020. Mild LOA will be the greatest cost of the LOA severities, increasing by 16% from 2020 to 2050, reaching an amount of £990.72 m (£985.97 m–£995.40 m) in 2050. Moderate LOA is estimated to see a slight decline in annual costs, falling from £275.22 m (£271.55 m–£278.95 m) in 2020 to £270.54 m (£262.38 m–£278.64 m) in 2050.

Our one-way sensitivity analysis revealed that total costs were most sensitive to the UDA contract value, and least sensitive to urgent rates across caries and periodontal pocketing Supplementary Figure S1.

## 4 Discussion

This study provides the first COI projection for direct NHS healthcare spending on oral diseases in the UK that has been built on national parameters, and provided cost estimates that are stratified by disease sub-type. The combination of using a previously published disease projection and top-down validation of our outputs increases confidence in our results. We produce markedly different estimates to the most recent projection by Botelho et al. (5). They estimate that direct costs attributable to periodontitis in the UK reached €120 m for 2018. We estimate that for 2020 periodontal pocketing and LOA will cost £3,186 m, however, these differences can be attributed to methodological differences. Botelho et al. (5) used national health expenditure data in a top-down approach that only captures expenditure that has been coded as periodontal treatment. This study assigned treatment costs to all individuals in a specific periodontal disease state, regardless of how their treatment is recorded. The top-down approach relies on condition-coded expenditure and will therefore underestimate the true economic burden of disease.

The key finding is the redistribution of the burden of costs towards older individuals (60 years and over) and towards more severe stages of oral diseases. Costs associated with untreated and treated caries in those aged 60 years and older are estimated to rise by 168% and 59%, respectively. This is a direct symptom of the projected increase in the UK adult population aged 60+, growing from 16.9 m to 22.5 m over the forecast period (2). The substantial burden of oral health in the older UK population has been previously established as an area which needs greater policy focus, and this provides economic rationale for that with our projections (14). Our findings support the view that the burden of oral disease in the UK will face an ageing-driven severity concentration, as we project the cost of treating severe periodontal pocketing and severe LOA to increase by 57% and 18%, respectively. This is due to the paradox that as individuals age they are more likely to consider severe oral health problems as insignificant due to the presence of diseases perceived as more urgent to treat (15). Individuals aged 60+ will bear 69% of caries-related costs by 2050. As a result, our projections help to build the economic case for shifting NHS dental resources towards the older adult oral health services which face greater barriers to access when compared to paediatric dental health services (16, 17).

Both the aged 16–24 and 25–59 treated and untreated caries costs are expected to fall throughout the forecast period. This has extenuated the increase in percent of total costs borne by 60+ year old, as we are seeing a pure increase in costs associated with treating older adults, while also witnessing a fall in the associated costs of those below 60 years old. A similar pattern is seen in urgent care, where the pace of growth in costs associated with those 60 years and older far outpaces the 25–59 age group, while the 16–24 age group actually sees a small decline in urgent costs for untreated caries.

The projected cost trajectory demonstrates a slow down in growth after 2035. We believe that this is due to a methodological flaw as opposed to a distinct epidemiological finding. This slow down reflects the structure of the disease prevalence projection used, which provides point estimates at 2020, 2035, and 2050. Therefore, our linear interpolation demonstrates an artifact of their modelling. This slow down is consistent with the rate of growth in the at-risk older population slowing after the post-war cohort.

UK dental policy for older adults has been previously described as too narrow due to its care home/institutional focus, possibly due to constrained treatment capacity due to the presence of competing diseases (18). This study provides the economic rationale that future policy needs to focus on taking the direction of the prevention of poor oral health over reactive treatment and intervention. We estimated that untreated caries will cost the NHS substantially more than treated caries. The active disease that is present in untreated caries requires greater restorative or extraction treatment (Band 2a/3 treatment), compared to the active monitoring and minor treatment that treated caries require (Band 1). Prevention is established as an important and achievable part of oral health policy, despite the increasing prevalence of untreated caries in the UK suggesting otherwise (19). One UK-based study recommends that a good patient–dentist relationship is key for individuals to engage in preventative behaviours through increased patient knowledge (20). Our estimations of the cost of untreated caries highlight that policy needs to focus on closing this information gap in order to reduce the burden on the NHS. There is an abundance of evidence on the use and effectiveness of behavioural preventative interventions in children in the UK, however, reviews have identified the lack of national policy priorities towards older adults (21, 22). This disparity in policy attention is unjustified given the large share of caries-related costs that will be borne by the 60+ group. Policy that integrates oral health assessments into wider health check-ups could provide a low cost way of reducing the projected economic burden, such as the Mouth Care Matters programme in the UK, which improved oral health provision in hospitals and produced health system savings (23).

It is important to benchmark our estimated direct healthcare costs to other prevalent diseases in the UK, as resource allocation in the NHS is competitive and bound by tight monetary and staffing constraints. Cardiovascular disease was estimated to have direct costs of £16.62b in the UK in 2021/2022 in a prevalence-based retrospective review (7). Using individual patient-level data, the costs for cancer, coronary heart disease, dementia and stroke were estimated to rise from 2018 to 2050 in England by 40%, 54%, 100%, and 85%, respectively (24). The direct costs of diabetes in 2021/2022 for the UK were estimated at £10.7b using population-level datasets (25). Annual chronic kidney disease costs for 2009/2010 were estimated at £1.44b–£1.45b through primary care and outpatient attendances (26). This places our estimates of direct NHS costs for 2030 of £4.83b, rising to £5.30b in 2050, as comparable to other substantial chronic diseases, exceeding NHS expenditure on chronic kidney disease, but more modest than the expected growth of dementia and stroke. Direct comparisons need to be treated with caution due to methodological differences and variety in cost perspectives taken. Our NHS estimate does not consider productivity loss, additional costs to patients, or quality-of-life impacts, which are included in estimates such as the £12.40b in stroke indirect costs (7). What can be deduced is that oral diseases will grow to be a substantial part of NHS expenditure, and that its primary driver, an ageing population, will also drive the growth in NHS expenditure of these other diseases.

Aside from the described absent societal perspective, our study has other limitations. We have used the NHS dental band system from England and applied it to the whole of the UK, despite small differences between the structure in Wales and Scotland, and this divergence is likely to increase as dental care reform takes place (27). Given our varying of UDA value through sensitivity analysis, we expect these differences to be mitigated, especially considering the small regional differences in payment amount and structure. Uniform attendance rates were assumed across the severities of LOA and periodontal pocketing due to a lack of available data on this. Similarly, urgent care attendance rates had to be derived from a combination of assumptions and estimations. We had to use linear interpolation to calculate the disease prevalence rates between given time points. While this should not largely affect the produced aggregate cost totals, it implies that our year-by-year specific estimations may lack precision. Costs are not discounted as we estimate annual burden and are not comparing interventions, which may limit comparisons to other disease areas where other projection methods are used. This framing characterises the real-terms resource flow associated with projected disease burden at each future time point rather than the present value of the projection horizon.

NHS dental planning needs to account for the growing disproportionate older adult treatment burden, and we have provided the economic rationale for preventive and early intervention. Future research needs to extend this model to measure what monetary and epidemiological effect an intervention could have on projected economic and disease burdens. Before the indirect and societal costs of oral diseases can be estimated and integrated into projections, greater research needs to be considered on the productivity loss and out-of-pocket expenditure associated with oral diseases.

## 5 Conclusion

This study provides projection estimates for the direct NHS costs of treating oral diseases from 2020 to 2050, offering the economic burden rationale for building preventative oral health policy in the UK. Future work needs to focus on quantifying the indirect and social cost at both an NHS and patient level to build a true burden estimate.

## Data Availability

The original contributions presented in the study are included in the article/Supplementary material, further inquiries can be directed to the corresponding author.
