# Supplementary material for: Estimating the direct healthcare costs of oral diseases in the United Kingdom: a cost-of-illness projection to 2050
Source: Front Public Health. 2026 Jun 12;14:1863227. doi: 10.3389/fpubh.2026.1863227 (PMC13303896; doi:10.3389/fpubh.2026.1863227)
Supplement: Supplementary file 1 [file Supplementary_file_1.docx]

**Supplementary Table S1:** Cost parameters, data sources, and justifications for the prevalence-based cost-of-illness model

*All costs in constant 2024/25 GBP (£). Effective annual cost per person = Cost per COT × Attendance rate × COTs per attendee per year.*

**Panel A: UDA contract value and NHS band cost structure**

| **Parameter** | **Value** | **UDAs** | **Cost per COT (£)** | **Source** |
| --- | --- | --- | --- | --- |
| Average UDA contract value | £31.00 | — | — | HFMA Briefing 2025 [10] |
| SA range | £28–£45 | — | — | NHS Dental Recovery Plan 2024; HFMA [10] |
| Band 1 (examination, prevention) | — | 1 | £31.00 | NHS BSA [11] |
| Band 2a (standard restorative) | — | 3 | £93.00 | NHS BSA [11] |
| Band 2b (non-molar endodontics) | — | 5 | £155.00 | NHS BSA [11] |
| Band 2c (molar endodontics) | — | 7 | £217.00 | NHS BSA [11] |
| Band 3 (crowns, dentures, bridges) | — | 12 | £372.00 | NHS BSA [11] |
| Urgent (emergency) | — | 1.2 | £37.20 | NHS BSA [11] |

**Panel B: State-specific annual planned treatment costs**

| **Disease state** | **Primary band** | **Cost per COT (£)** | **Attendance rate (%)** | **COTs per year** | **Effective annual cost (£)** | **Source / Justification** |
| --- | --- | --- | --- | --- | --- | --- |
| ***Caries states*** |  |  |  |  |  |  |
| No caries | Band 1 | £31.00 | 51.0% | 1 | £15.81 | Band 1 check-up only. Attendance: AOHS 2023 (51% regular check-up) [7]. |
| Untreated caries 16–24 | Band 2a/3 mix | £120.90 | 52.1% | 1 | £62.99 | Weighted average Band 2a (£93) + Band 3 (£372). Higher extraction rate in young adults. Attendance: AOHS 2023 Table 2 (16–24: 52.1%) [7]. |
| Untreated caries 25–59 | Band 2a dominant | £106.95 | 52.0% | 1 | £55.61 | Primarily fillings + extractions, lower Band 3 weighting than 60+. Attendance: AOHS 2023 (25–59: 52.0%) [7]. |
| Untreated caries 60+ | Band 2a/3 mix | £162.75 | 68.0% | 1 | £110.67 | Higher prosthetic need; greater Band 3 weighting. Attendance: AOHS 2023 (60+: 68.0%) [7]. |
| Treated caries 16–24 | Band 1 | £31.00 | 52.1% | 1 | £16.15 | Sound restorations — monitoring only (Band 1). Attendance assumed same as untreated (limitation). AOHS 2023 [7]. |
| Treated caries 25–59 | Band 1 | £31.00 | 52.0% | 1 | £16.12 | Sound restorations — monitoring only. Attendance: AOHS 2023 [7]. |
| Treated caries 60+ | Band 1 | £31.00 | 68.0% | 1 | £21.08 | Sound restorations — monitoring only. Attendance: AOHS 2023 [7]. |
| ***Periodontal states*** |  |  |  |  |  |  |
| No periodontal condition | Band 1 | £31.00 | 58.0% | 1 | £17.98 | Band 1 check-up only if attending. Attendance: AOHS 2023 overall annual rate (58%) [7]. No age split available for perio states (limitation). |
| Mild pocketing (4–<6mm) | Band 2a | £93.00 | 58.0% | 1 | £53.94 | Basic periodontal treatment (scale & polish, RSD). Attendance: AOHS 2023 overall (58%) [7]. |
| Moderate pocketing (6–<9mm) | Band 2a | £93.00 | 58.0% | 1 | £53.94 | Intensive periodontal treatment or specialist referral pathway. Same UDA cost as mild within the NHS band structure. Attendance: AOHS 2023 (58%) [7]. |
| Severe pocketing (≥9mm) | Band 2a/3 mix | £232.50 | 58.0% | 2 | £269.70 | Weighted average Band 2a + Band 3 reflecting complex treatment, extraction of hopeless teeth, and prosthetic replacement. 2 COTs/yr for staged treatment. Attendance: AOHS 2023 (58%) [7]. |
| ***Loss of attachment states (55+ only)*** |  |  |  |  |  |  |
| Mild LOA (4–<6mm) | Band 2a | £93.00 | 68.0% | 1 | £63.24 | Periodontal maintenance. LOA assessed in 55+ only. Attendance: AOHS 2023 (60+: 68%) [7]. |
| Moderate LOA (6–<9mm) | Band 2a | £93.00 | 68.0% | 1 | £63.24 | Periodontal maintenance. Attendance: AOHS 2023 (60+: 68%) [7]. |
| Severe LOA (≥9mm) | Band 2a/3 mix | £232.50 | 68.0% | 2 | £316.20 | High extraction + prosthetic cost. Same band mix and COT logic as severe pocketing. Attendance: AOHS 2023 (60+: 68%) [7]. |

**Panel C: Urgent care module — annual emergency presentation rates by disease state**

*Urgent care is not a separate state in the Elamin & Ansah model. A proportion of each disease-state population presents as an emergency per year. Urgent cost per episode = 1.2 UDAs × £31 = £37.20.*

| **Disease state** | **Annual urgent presentation rate** | **Urgent cost per episode (£)** | **Effective annual urgent cost (£)** | **Source / Justification** |
| --- | --- | --- | --- | --- |
| No caries / no perio | 2.0% | £37.20 | £0.74 | Estimated 2%. Minimal emergency risk for disease-free population. Trauma/other causes only. |
| Untreated caries 16–24 | 4.7% | £37.20 | £1.75 | AOHS 2023 Table 6: prevalence of potentially urgent conditions ~4.7% (16–24) [7]. |
| Untreated caries 25–59 | 20.0% | £37.20 | £7.44 | AOHS 2023 Table 6: average 25–54 ≈ 19–22% [7]. Pulpitis, abscess. |
| Untreated caries 60+ | 22.0% | £37.20 | £8.18 | AOHS 2023 Table 6: average 65–75+ ≈ 20–23% [7]. Abscess, fractured teeth. |
| Treated caries (all ages) | 5.0% | £37.20 | £1.86 | Estimated 5%. Lower than untreated — failed restoration, debond, fracture. |
| Mild pocketing (4–<6mm) | 3.0% | £37.20 | £1.12 | Estimated 3%. Periodontal abscess rare at mild severity. |
| Moderate pocketing (6–<9mm) | 8.0% | £37.20 | £2.98 | Estimated 8%. Periodontal abscess and acute episodes possible. |
| Severe pocketing (≥9mm) | 15.0% | £37.20 | £5.58 | Estimated 15%. Periodontal abscess, pain, swelling, tooth mobility. |
| Mild LOA (4–<6mm) | 3.0% | £37.20 | £1.12 | Estimated 3%. Similar to mild pocketing. |
| Moderate LOA (6–<9mm) | 8.0% | £37.20 | £2.98 | Estimated 8%. Similar to moderate pocketing. |
| Severe LOA (≥9mm) | 15.0% | £37.20 | £5.58 | Estimated 15%. Tooth mobility, acute episodes. |

**Panel D: Derivation of attendance rates from AOHS 2023**

*AOHS 2023 Table 2 reports frequency of dental attendance. "At least annual" = six-monthly + annual attenders. Rates mapped to Elamin & Ansah age bands (16–24, 25–59, 60+).*

| **AOHS 2023 age group** | **Six-monthly (%)** | **Annual (%)** | **At least annual (%)** | **Mapped model rate** |
| --- | --- | --- | --- | --- |
| 16–24 | 36.2% | 15.9% | 52.1% | → 16–24: 52.1% |
| 25–34 | 21.2% | 21.5% | 42.7% |  |
| 35–44 | 32.4% | 22.3% | 54.7% |  |
| 45–54 | 32.6% | 26.3% | 58.9% | → 25–59: 52.0% |
| 55–64 | 47.1% | 15.7% | 62.8% | (weighted average) |
| 65–74 | 54.4% | 18.5% | 72.9% |  |
| 75+ | 52.0% | 15.7% | 67.7% | → 60+: 68.0% |
| All adults | 38.2% | 19.8% | 58.0% | → Perio overall: 58.0% |

**Key assumptions and limitations:**

*1. Attendance rates applied uniformly across disease states within each age group (AOHS does not report attendance by disease state).*

*2. AOHS 2023 age bands do not perfectly align with Elamin & Ansah age bands (16–24, 25–59, 60+); mapping is approximate.*

*3. Urgent presentation rates for periodontal states are estimated from clinical judgement; AOHS Table 6 reports caries-related urgent conditions only.*

*4. Treated caries assumed same attendance rate as untreated caries; treated patients may attend more frequently (selection effect).*

*5. Periodontal state attendance uses overall rate (58%) as no severity-specific attendance data available from AOHS.*

*6. Costs held constant in real terms at 2024/25 levels across the 2020–2050 projection horizon.*

*7. Band 2 sub-bands (2a, 2b, 2c) introduced November 2022; untreated caries costs use weighted averages across sub-bands.*

**Panel E: Band-mix weightings for disease states requiring mixed-band treatment**

*Disease states where treatment spans multiple NHS bands have costs calculated as weighted averages. Band 2a (3 UDAs, £93.00) covers standard restorative treatment (fillings, extractions, root surface debridement). Band 3 (12 UDAs, £372.00) covers complex laboratory work (crowns, dentures, bridges). All costs are derived from the UDA contract value of £31.00 and are therefore fully responsive to changes in this parameter.*

| **Disease state** | **Band 2a weight** | **Band 3 weight** | **Weighted avg cost per COT** | **Effective annual cost per person** | **Clinical justification for weighting** |
| --- | --- | --- | --- | --- | --- |
| Untreated caries 16–24 | 90% | 10% | £120.90 | £62.99 | Young adults with active caries primarily receive fillings and extractions (Band 2a). The 10% Band 3 weighting reflects occasional prosthetic need following multiple extractions. This is the lowest Band 3 weighting of the three age groups because younger patients have fewer accumulated extractions and lower denture prevalence (ADHS 2009: 0% edentulousness in 16–24 vs 15% in 65–74). |
| Untreated caries 25–59 | 95% | 5% | £106.95 | £55.61 | Working-age adults with untreated caries predominantly require restorative treatment. The 5% Band 3 weighting — lower than 16–24 — reflects the expectation that this group retains more teeth and has greater access to restorative care (fillings, root canal) rather than extraction and replacement. The ADHS 2009 shows lower edentulousness rates in 25–44 (0%) and 45–54 (1%) than in older groups. |
| Untreated caries 60+ | 75% | 25% | £162.75 | £110.67 | Older adults with untreated caries have the highest prosthetic need. The 25% Band 3 weighting reflects accumulated tooth loss, root caries on exposed surfaces, and a higher proportion of unrestorable teeth requiring extraction and prosthetic replacement. ADHS 2009 shows edentulousness rising from 6% (55–64) to 15% (65–74) to 30% (75–84), supporting a substantially higher Band 3 weighting in this group. |
| Severe pocketing (≥9mm) | 50% | 50% | £232.50 | £269.70 | Severe periodontal disease involves a dual treatment pathway: intensive non-surgical periodontal therapy including staged root surface debridement (Band 2a), and extraction of teeth with hopeless prognosis followed by prosthetic replacement (Band 3). The equal 50/50 weighting reflects clinical consensus that approximately half of patients with ≥9mm pocketing will require prosthetic rehabilitation within a treatment cycle. The effective annual cost also reflects 2 COTs per year for staged treatment. |
| Severe LOA (≥9mm) | 50% | 50% | £232.50 | £316.20 | Same treatment pathway rationale as severe pocketing. Advanced attachment loss in the 55+ population leads to equal need for intensive periodontal treatment and prosthetic replacement. The higher effective annual cost compared to severe pocketing (£316.20 vs £269.70) reflects the higher attendance rate applied to LOA states (68% for 60+ vs 58% overall for periodontal states). |

**Sensitivity analysis implications:** *All band-mix weightings are editable in the model. Since the UDA contract value (£31) is the single most influential parameter (£2,907m swing in one-way SA), and all band costs are derived from this value, the band-mix proportions interact multiplicatively with the UDA value. Varying the Band 3 weighting for untreated caries 60+ from 15% to 35% (±10 percentage points from base case) would change the effective annual cost from £96.79 to £124.55, representing a ±13% variation — substantially smaller than the ±45% swing from the UDA value itself (£28–£45 range). This supports the prioritisation of UDA value uncertainty over band-mix uncertainty in the sensitivity analysis.*

**States with single-band assignment (no weighting required):** *No caries and treated caries (all ages) = Band 1 only (£31.00). No periodontal condition = Band 1 only (£31.00). Mild and moderate pocketing = Band 2a only (£93.00). Mild and moderate LOA = Band 2a only (£93.00). These assignments are unambiguous within the NHS band structure: Band 1 covers examinations and preventive care; Band 2a covers standard restorative and periodontal treatment.*

**Figure S1**: Results of one-way sensitivity analysis
